# Integrated Multifunctional Graphene Discs 2D Plasmonic Optical Tweezers for Manipulating Nanoparticles

**DOI:** 10.3390/nano12101769

**Published:** 2022-05-23

**Authors:** Hongyan Yang, Ziyang Mei, Zhenkai Li, Houquan Liu, Hongchang Deng, Gongli Xiao, Jianqing Li, Yunhan Luo, Libo Yuan

**Affiliations:** 1 College of Optoelectronic Engineering, Guilin University of Electronic Technology, Guilin 541004, China; hyyang@guet.edu.cn (H.Y.); 19082203004@mails.guet.edu.cn (Z.M.); 19082203009@mails.guet.edu.cn (Z.L.); liuhouq@guet.edu.cn (H.L.); cdeng@guet.edu.cn (H.D.); lbyuan@guet.edu.cn (L.Y.); 2 Guangxi Key Laboratory of Optoelectronic Information Processing, Guilin University of Electronic Technology, Guilin 541004, China; 3 Guangxi Key Laboratory of Precision Navigation Technology and Application, Guilin University of Electronic Technology, Guilin 541004, China; 4 Guangdong-Hong Kong-Macao Joint Laboratory for Intelligent Micro-Nano Optoelectronic Technology, Macau University of Science and Technology, Macau 999078, China; jqli@must.edu.mo; 5 College of Science & Engineering, Jinan University, Guangzhou 510632, China; luoyunhan@jnu.edu.cn

**Keywords:** plasmonic optical tweezers, graphene, optical manipulation

## Abstract

Optical tweezers are key tools to trap and manipulate nanoparticles in a non-invasive way, and have been widely used in the biological and medical fields. We present an integrated multifunctional 2D plasmonic optical tweezer consisting of an array of graphene discs and the substrate circuit. The substrate circuit allows us to apply a bias voltage to configure the Fermi energy of graphene discs independently. Our work is based on numerical simulation of the finite element method. Numerical results show that the optical force is generated due to the localized surface plasmonic resonance (LSPR) mode of the graphene discs with Fermi Energy E*_f_* = 0.6 eV under incident intensity I = 1 mW/μm^2^, which has a very low incident intensity compared to other plasmonic tweezers systems. The optical forces on the nanoparticles can be controlled by modulating the position of LSPR excitation. Controlling the position of LSPR excitation by bias voltage gates to configure the Fermi energy of graphene disks, the nanoparticles can be dynamically transported to arbitrary positions in the 2D plane. Our work is integrated and has multiple functions, which can be applied to trap, transport, sort, and fuse nanoparticles independently. It has potential applications in many fields, such as lab-on-a-chip, nano assembly, enhanced Raman sensing, etc.

## 1. Introduction

Since Ashkin first introduced the concept of optical tweezers through experiments and theoretical works in the 1970s [1,2], this technology has gained widespread application in the biological and medical fields with its ability to trap and manipulate nanoparticles in a non-invasion way [3,4,5,6]. Conventional laser optical tweezers based on tightly focused laser beams are powerful tools for trapping metal nanowires [7], dielectric particles [8], proteins [9], viruses [3], and cells [10], and have been widely and intensively studied. The particle trapping can be achieved by balancing the gradient force and scattering force on the particles through the strong electric field gradient formed by the focused lens laser beams [1]. However, due to the diffraction limit, the focal size of the focused beams cannot be smaller than the incident light wavelength, which greatly limits the application of focused laser beams to trap nanoparticles [2,11]. To obtain greater trapping stiffness to overcome Brownian motion, conventional focused beam tweezers need to induce high-power laser light to increase the electric field gradient. However, high-power incident light can potentially cause optical damage and thermal damage to biological nanoparticles [12,13]. Plasmonic optical tweezers with artificial micro-nano antennas are an effective solution to overcome the diffraction limit and eliminate the influence of the photothermal effect [14,15,16]. The plasmonic antenna is generally composed of noble metal structures, including double nano-aperture [17], dipole antennas [18], nano-circular holes [19], etc. When the incident light frequency matches the overall resonant frequency of the system, it can absorb a large amount of incident light energy to generate optical hot spots with extremely sharp electric field gradients on the metal surface, forming a plasmonic resonance [20,21,22]. When nanoparticles approach the plasmonic antenna with Brownian motion, the nanoparticle will be subjected to a strong optical force pointing to the place of maximum electric field strength. The distribution of trapping potential and optical forces generated by plasmonic optical tweezers are generally determined after fabrication, therefore they cannot achieve dynamic manipulation of nanoparticles, which limits the practical application of plasmonic optical tweezers [23,24]. Then some remarkable ideas have been proposed. By dynamically modulating the characteristics of the incident light (phase [25], polarization [26,27,28], intensity [24], wavelength [29], the excitation position of the plasmonic resonance can be switched, and thus trapping positions can also be changed together. The switching of adjacent trapping positions is the key to these schemes. Optoelectronic tweezers can also effectively eliminate the influence of diffraction limit and photothermal effect, and introduce physical electrode control, which is more flexible in design and function [30,31].

Graphene is a new two-dimensional material with a single-layer carbon atom structure. Due to its zero bandgaps and unique photoelectric properties, graphene has gained much attention in recent years due to its unique optical properties [32,33,34,35,36]. Its electrical conductivity can be adjusted by an electric gate and chemical doping [37,38]. Graphene is gradually becoming a hot spot for research on planar tunable metamaterials. Graphene can be processed into different shapes, such as nanoribbons [39,40], nanodisks [41,42], nanohole [43,44]. Plasmonic optical tweezers based on graphene have been proposed and demonstrated in the past few years. Bofeng Zhu’group first proposed a giant gradient force for nanoparticle trapping in coupled graphene strips waveguides in 2015 [45]. More recently, Jianfa Zhang’s group proposed a graphene circular hole that can stably trap 10 nm particles under moderate incident intensity [46], Mohsen Samadi’s group proposed particle sorting based on double-striped graphene [47], and Peter Qiang Liu’s group proposed a plasmonic transport network with graphene strips [23]. All of these works are achieved by using a bias voltage to control the Fermi of graphene to control the optical force and achieve specific functionality. Obvious, it is still a big challenge to obtain multifunctional integrated plasmonic optical tweezers and manipulate nanoparticles in a two-dimensional plane without a fixed track.

Here we theoretically propose integrated multifunctional graphene discs 2D plasmonic optical tweezers for manipulating nanoparticles, which can trap and transport nanoparticles anywhere in the 2D plane by applying bias voltage without a preset path. Our work is based on numerical simulation of the finite element method. The distribution of optical forces on the nanoparticles and the trapping potential are simulated theoretically using the finite element method. Further, we perform simulations of the photothermal fluid and estimate the magnitude of other major forces on the nanoparticle, including the Brownian motion force, the Stokes’s drag force, and thermophoretic force. We numerically demonstrate the ability of this system to trap, transport, and fuse nanoparticles through the Langevin equation, and plot the motion trajectory of the nanoparticles. This work can be applied to trapping, transporting, sorting, and fusion of nanoparticles flexibly and has potential applications in many fields, such as optical sensing, enhanced Raman sensing, biophysics, Opto-mechanic, etc.

## 2. Structure Design and Modeling

**Figure 1:**
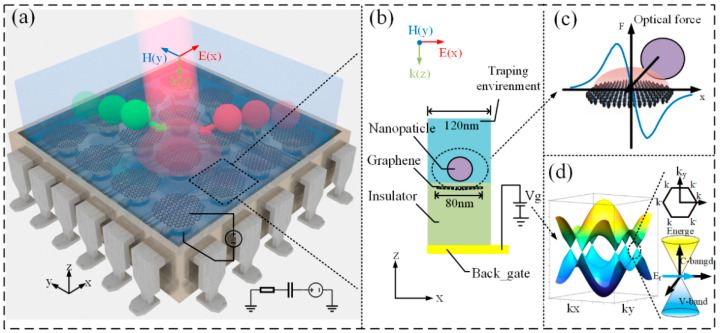
(**a**) 3D schematic diagram of Tunable graphene disks 2D plasmonic tweezers for trapping and transportation of nanoparticles. (**b**) Schematic of the cross-section of single graphene disc structure for optical trapping of nanoparticles. (**c**) MST optical forces on a dielectric nanosphere. (**d**) Band structure of graphene electrodes under a voltage bias.

Figure 1a shows the schematic of integrated multifunctional graphene discs 2D plasmonic optical tweezers for manipulating nanoparticles. The schematic of the cross-section of a single graphene disk is shown in Figure 1b. The graphene disc is set as 10 nm below the interface of the trapping environment and the gate insulator. This can isolate the graphene disk from the trapping environment. The back gate is used to modulate the Fermi energy of graphene discs. The circuit under the graphene discs array allows us to modulate the Fermi energy of anyone graphene disk independently. The conversion of graphene from n-type to p-type reference can be achieved by applying a bias voltage as shown in Figure 1d. The system is surrounded by water (*n* = 1.33) under a normal incident plane wave with an intensity of 1 mW/µm^2^. The material of the insulator substrate layer is designated as BaF_2_ (*n* = 1.45), which has high transmittance in the mid-IR and has been proven to be applicable in graphene optical tweezer systems [23]. We choose polyethylene nanoparticles for research because dielectric nanoparticles are widely used in the biological and medical fields [48]. When the Fermi energy of a single graphene disk is 0.6eV, the component of the optical force on the particle (radius 50 nm refractive index 1.6, positioned at 20 nm above the interface) is displayed in Figure 1c. Continuous monolayer graphene can be generated using the chemical vapor deposition (CVD) technique and transferred onto the substrate by the graphene transfer method [49]. Monolayers of graphene layers can be patterned into graphene disk arrays using focused ion-beam (FIB) [50]. The challenge of processing is to deposit a fixed thickness of the insulating layer over the graphene disk on the substrate.

We perform a full 3D simulation using a finite element method (COMSOL Multiphysics). In our numerical study, Graphene is modeled as a two-dimensional transaction boundary condition with a thickness of 0.34 nm [51]. The Kubo model is used to describe the conductivity, which takes the intra-band and inter-band transmission into account as [52]:

(1) $$\begin{array}{l} \sigma_{gra}=\sigma_{\mathrm{int}ra}+\sigma_{\mathrm{int}er}=\frac{2e^{2}k_{B}T}{\pi \hbar^{2}}\frac{i}{\omega +i/\tau }\ln[2\cosh(\frac{E_{f}}{2k_{B}T})]+\frac{e^{2}}{4\hbar^{2}}[\frac{1}{2}\\ +\frac{1}{\pi }\arctan(\frac{\hbar \omega -2E_{f}}{2k_{B}T})-\frac{i}{2\pi }\ln\frac{{\left(\hbar \omega +2E_{f}\right)}^{2}}{{\left(\hbar \omega -2E_{f}\right)}^{2}+4{\left(k_{B}T\right)}^{2}}] \end{array}$$

Here $k_{B}$, e, and ℏ are Boltzmann constant, electron charge, and reduced Plank constant, respectively. T is the temperature of the environment. ω is the angular frequency of incident light, $E_{f}$ is the Fermi energy of graphene. τ = 1 ps is the electron-phonon relaxation time. The gate voltage required to regulate the Fermi level of graphene disk can be calculated by [53]:

(2) $$V_{g}=\frac{t_{d}}{\varepsilon_{0}\varepsilon_{d}}\cdot \frac{E_{f}^{2}}{\pi \hbar^{2}v_{f}^{2}}-V_{0}$$

Here ε_0_ and ε*_d_* are the dielectric constant and the thickness of the gate oxide. $V_{0}$ is the offset voltage caused by natural doping. $v_{f}$ ≈ 10^6^ m/s is Fermi velocity. *t_d_* is the distance from the graphene disc to the bias voltage gate. We use the Maxwell stress tensor to calculate the time-averaged optical force on the nanoparticle [54,55].

(3) 〈F〉=∫S12Re〈T〉⋅ndS

Here **S** is the outer surface of the nanoparticle and **n** is its discrete normal vector, 〈〉 represents the time average. 〈*T*〉 is the Maxwell stress tensor which can be governed as:

(4) 〈T〉=εE

Here ε, μ are the permittivity and permeability close to the nanoparticle surface in the trapping environment. **E** and **H** are the intensity vectors of the electric and magnetic fields on the surface of the particle, respectively. Through Helmholtz Hodge decomposition, the optical force can be decomposed into conservative force and non-conservative force. We only take the conservative force into consideration [56,57]. The trapping potential in each direction can be approximately calculated as the line integral of the component of the optical force on the nanoparticle [58]:

(5) $$U_{r}=-\int_{-\infty }^{r}F(r\prime )dr\prime$$

## 3. Results and Discussion

**Figure 2:**
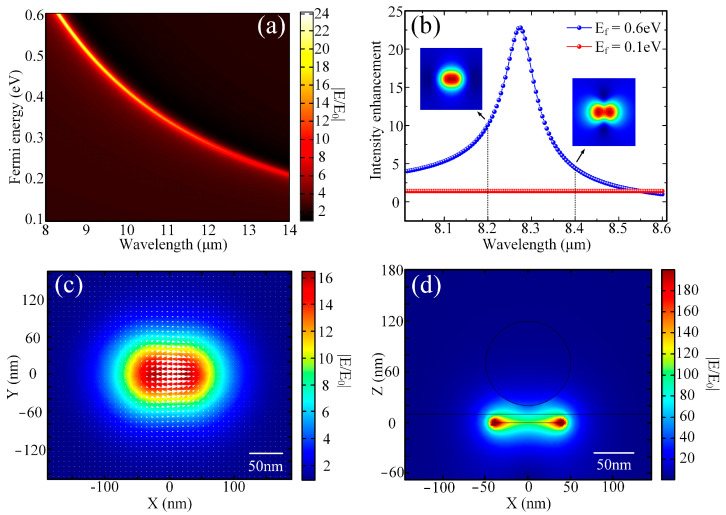
(**a**) The normalized intensity enhancement map under different Fermi energy and incident wavelengths. (**b**) The normalized intensity enhancement under different Fermi energy (E*_f_* = 0.1 eV, E*_f_* = 0.6 eV), and corresponding simulated amplitude distribution. (**c**) Simulated amplitude distribution in the 50 nm x-y plane above the graphene disc. The arrow (white) indicates the direction of the current. (**d**) Simulated amplitude distribution in the x-z plane after the introduction of the nanoparticle.

It is well known that plasmonic systems with different structures have specific resonance wavelengths. To better understand the resonance distribution of a graphene disc under incident light, We set up a probe at 50 nm above the center of the graphene disc (the center of the nanoparticle) to determine the efficiency of near-field enhancement. A normalized intensity map is represented in Figure 2a. As is shown in the picture, the electric field intensity at the probe can be enhanced by more than 24 times compared to the intensity of the incident light. The localized surface plasmon resonance (LSPR) intensity of graphene is enhanced at a specific wavelength and Fermi energy. The resonance wavelength blue shifts when Fermi energy decreases. Further, we investigate the resonance of graphene discs as the Fermi energy is 0.1 eV and 0.6 eV. The normalized intensity map of graphene disk plasmonic resonance for two different Fermi energy (0.6 eV, 0.1 eV) versus incident light wavelengths is shown in Figure 2b. It can be observed that the plasmonic resonance of the graphene disc under the incident light is less than two times enhanced when the Fermi energy is 0.1 eV, and more than 24 times enhanced when the Fermi energy of 0.6 eV. When the Fermi energy is 0.6 eV, the strongest resonance wavelength of the graphene disk is 8.28 nm, and two resonance modes are formed on both sides of it. When the wavelength of the incident light is 8.2 μm, the intensity enhancement of the plasmonic resonance is concentrated at the center of the graphene disk. When the wavelength of the incident light is more than 8.4 μm, the intensity enhancement of the plasmonic resonance is dispersed at the center of the graphene discs. Comparing these two resonance modes, the resonance mode when the wavelength of the incident light is 8.26 μm exhibits the potential for trapping nanoparticles in the center. We set the wavelength of the incident light as 8.26 μm. The normalized electric field intensity of the resonance mode (50 nm above the graphene disk) is shown in Figure 2c. Next, we introduce a spherical nanoparticle (radius of 50 nm and refractive index of 1.6) in our simulation. The center position of this spherical nanoparticle is located at x-center = 0 nm, y-center = 0 nm and z-center = 70 nm, respectively. The results are shown in Figure 2d. Part of the nanoparticle is contained in the near-field enhancement of the plasmonic resonance. This phenomenon exhibits the potential to trap nanoparticles.

**Figure 3:**
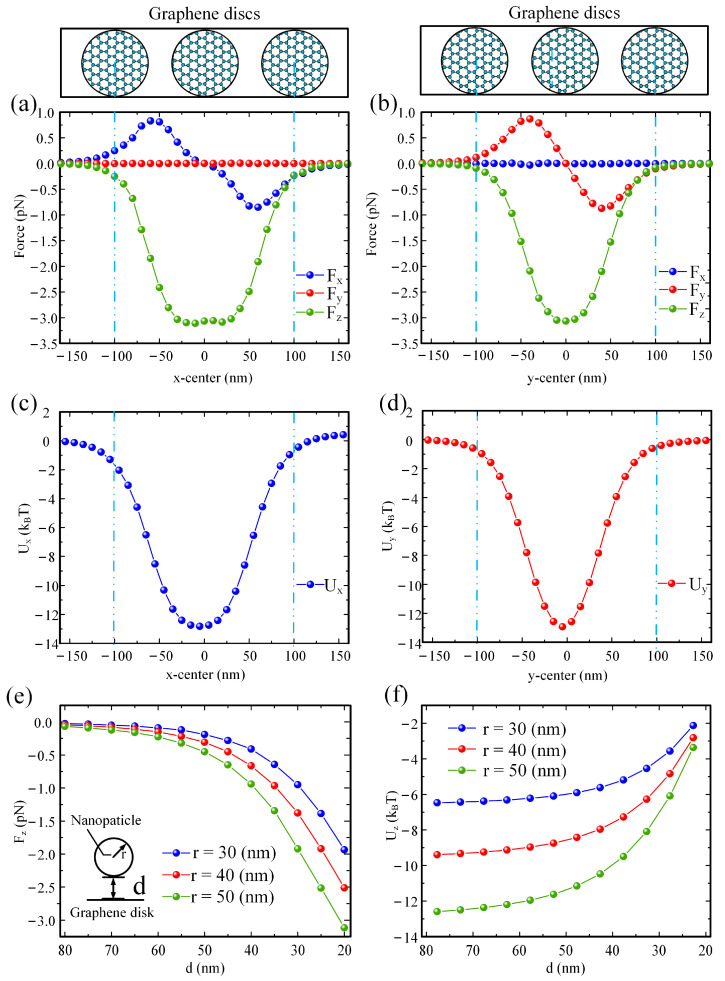
The components of the optical force on the nanoparticle (*n* = 1.6) and the corresponding trapping potential are calculated by the MST method. (**a**,**c**) The center of the nanoparticle is on the *x*-axis. (**b**,**d**) The center of the nanoparticle is on the *y*-axis. (**e**,**f**) The center of the nanoparticle is in the *x*-axis, z-center, r = 30, 40, and 50 nm, respectively.

We continue to investigate the optical forces on the nanoparticle (radius 50 nm, refractive index 1.6) at different locations, Fermi energy of the graphene disc is configured as 0.6 eV. We assume that the intensity of the incident light is I_0_ = 1 mW/µm^2^, which has much lower than other plasmonic optical tweezer systems [42,43]. The optical forces on the nanoparticles and the trapping potential are calculated by the time-averaged tensor (Equations (3) and (4)) and the line integral calculation (Equation (5)), respectively. Figure 3a shows the components of the optical force as a function of the nanoparticle centered along the *x*-axis, with y-center = 0 and z-center = 50 nm. When x-center = −160 nm~160 nm, F_z_ is always negative (F_z_ < 0 pN), which proves that the nanoparticle will be pulled to the interface between the dielectric material and the trapping environment. When x-center > 0, F_x_ is a positive force (F_x_ > 0 pN), x-center < 0, F_x_ is a negative force (F_x_ < 0 pN), x-center = 0, F_x_ = 0 pN. This means that the nanoparticle will be trapped by the optical force in the center of the graphene disk along the *x*-axis. As the result shown in Figure 3c, by the line integral of the force along the *x*-axis, we calculate the trapping potential of the nanoparticle along the *x*-axis, which is greater than 10 K_B_T to ensure stable trapping. Corresponding to the graphene disks diagram at the top of the figure, the trapping region in the *x*-axis direction contains the entire area of the adjacent graphene discs. This demonstrates that, by dynamically configuring the Fermi level of graphene, the motion of nanoparticles can be controlled along the *x*-axis. Figure 3b shows the components of the optical force as a function of the nanoparticle centered along the *y*-axis, with x-center = 0 nm and z-center = 50 nm. F_y_ is a positive force (F_y_ > 0 pN), y-center < 0, F_y_ is a negative force (F_y_ < 0 pN), y-center = 0, F_y_ = 0 pN. This means that the nanoparticle will be trapped by the optical force in the center of the graphene disk along the y-axis. As the result shown in Figure 3d, by the line integral of the force along the *y*-axis, we calculate the trapping potential of the particle along the *y*-axis, which is greater than 10 K_B_T to ensure stable trapping. Corresponding to the graphene disks diagram at the top of the figure, the trapping region in the *y*-axis direction contains the entire area of the adjacent graphene discs. This demonstrates that, by dynamically configuring the Fermi level of graphene, the motion of nanoparticles can be controlled along the *y*-axis. Figure 3e shows optical forces Fz on nanoparticles with different radius radii as the nanoparticle centered along the *z*-axis, with x-center = 0 nm and y-center = 0 nm. d is the distance from the bottom of the nanoparticle to the graphene disc. It can be found that the larger the radius of the nanoparticle the greater the optical force on it. By the line integral of the force along the *z*-axis, we calculate the trapping potential of the nanoparticle on the *x*-axis. The result is shown In Figure 3f. When the radius of the nanoparticle is less than 50 nm, the trapping potential is less than 10 K_B_T. This can be improved by appropriately increasing the incident light intensity.

**Figure 4:**
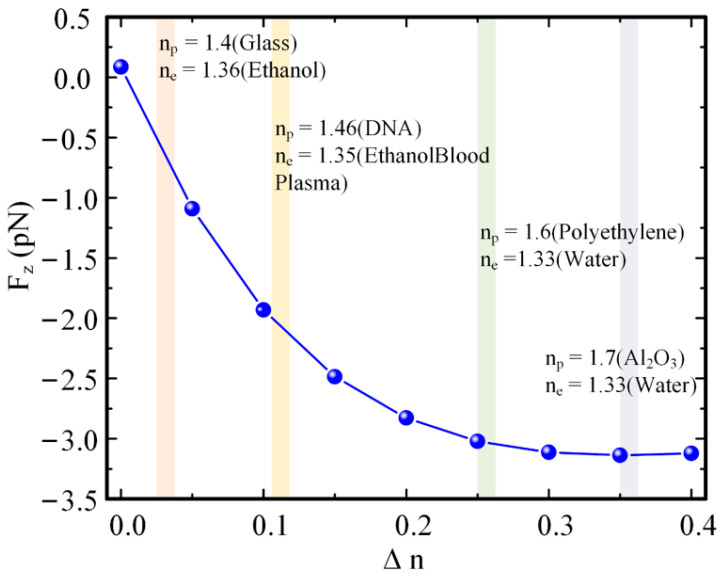
The optical forces on nanoparticles of different materials in different environment materials, Δn = n_p_ − n_e_. n_p_ and n_e_ are the refractive indices of the nanoparticles and the trapped environment, respectively.

Figure 4 discusses the suitability (different values of the refractive index of nanoparticles and trapping environments) of the system. The optical forces on nanoparticles of different materials in different trapping environments are shown in Figure 4. The center of a nanoparticle is fixed at the position of the center of the graphene disk, and the bottom of the nanoparticle is 20 nm above the graphene disk. The larger the refractive index difference between the nanoparticle and the tapping environment, the greater the optical force on the nanoparticle. However, the growth is gradually slowing down because the high refractive index nanoparticles weaken the strength of LSPR of the graphene discs. To achieve a stable tapping of nanoparticles, we can appropriately increase the intensity of the incident light.

**Figure 5:**
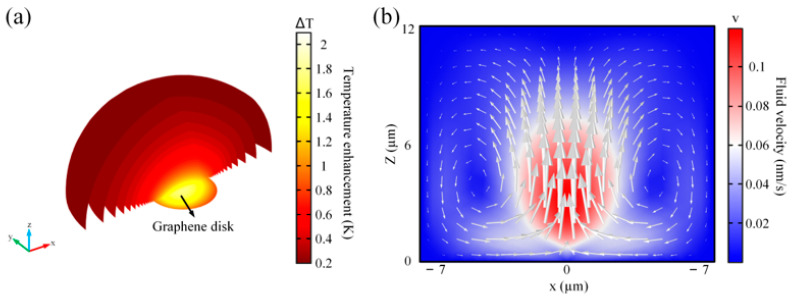
(**a**) Heat power dissipation density Around the graphene disk, (**b**) the steady-state fluid velocity and fluid vector under the incident light intensity I_0_ = 1 mW/µm^2^.

We next numerically investigate the temperature distribution and convection field near the graphene disc (E*_f_* = 0.6 eV) under an incident light intensity I_0_ = 1 mW/µm^2^. Figure 5a shows the temperature distribution. Graphene discs generate less electromagnetic heat than metallic materials because of their two-dimensional molecular composition (with a thickness of 0.34 nm) and high thermal conductivity ($\kappa_{\mathrm{Graphene}}=2000\;WK^{-1}m^{-1},\kappa_{\mathrm{Au}}=314\;WK^{-1}m^{-1}$) at room temperature [59]. As is shown in Figure 5a, maximum rise temperature ΔT = T_s_ − T_e_. T_s_ is the temperature at each point when the simulation reaches a steady state. T_e_ is the operating room temperature. ΔT increases only 2 °C compared to room temperature. To estimate the thermal behavior in the proposed system, The thermally induced convective distribution is represented in Figure 5b, including the fluid amplitude distribution (color map) and the fluid vector (arrows). The maximum convection velocity vectors can reach v = 0.1 nm/s. Flowing water is a radial symmetrical cycle that travels outward and upward. Driven by gravity and buoyancy, a Rayleigh-Benard system is formed [60,61]. This can improve trapping efficiency and prevent particles from sticking to the graphene disk.

Using the data in Figure 5, we further estimate the forces on the particle, including the optical force, Brownian motion force, Stokes’ drag force, thermophoretic force, and gravity. The calculation equations and results are expressed in Table 1 [47,62,63]. γ=6πηr is the viscous drag coefficient given by Stokes’s law η = 88.9 × 10^−5^ ${\mathrm{sm}}^{-1}$ is the dynamic viscosity of the water. The convective force on the nanoparticle can be described by Stokes’ drag force equation, in which v is the fluid convection velocity vector. The thermophoretic force on the nanoparticle in the temperature gradient can be calculated using the formula in the table. ∇T, $D_{T}$, are the thermal field gradient and thermophoretic mobility at a steady-state, respectively. For nanoparticles with a radius of 50 nm and a refractive index of 1.6 (polyethylene) in water, we assume $\nabla T\approx 55.6\;K\mu m^{-1}$, $D_{T}\approx 1.55\;\mu m^{2}s^{-1}$ [43]. We also evaluate the gravity force of the nanoparticle assuming the density of $\rho =950\;\mathrm{kg}/m^{3}$ (polythene). As the results are shown in Table 1. The other kinds of forces on the nanoparticle are less than two or three orders of magnitude compared to the optical force during trapping time, and they have no significant effect on the trapping process of our optical tweezers.

Next, how to achieve the function of trapping, transporting, and fusing nanoparticles in the 2D plane by modulating the Fermi energy of graphene discs through the back gate will be demonstrated. In our design, we will configure the Fermi energy of graphene discs as 0.6 eV for the ON state and 0.1 eV for the OFF state under the incident intensity I_0_ = 1 mW/µm^2^. Through the previous discussion, the graphene disk in the ON state can produce strong plasmonic resonance to trap the nanoparticles in the middle, but the OFF state cannot be enhanced. The function of the system is achieved by switching the graphene disc ON and OFF states. Here we use a simplified Langevin equation for the simulation [64]:

(6) $$r(t)=r(t-\Delta t)+\frac{\Delta t}{\gamma }F(t)+\sqrt{\frac{2k_{B}T\Delta t}{\gamma }}•W(t)$$

where r(t) is the position of the center of the nanoparticle at moment t. Δt, K_B_, and T are the time step, Boltzmann’s constant, and the temperature of the environment, respectively. W(t) is a vector of Gaussian random numbers, whose average is 0 and its variance is 1. To simplify the calculation, the optical force is calculated by the dipole method, with the nanoparticle approximated as an electric dipole [55,63].

**Figure 6:**
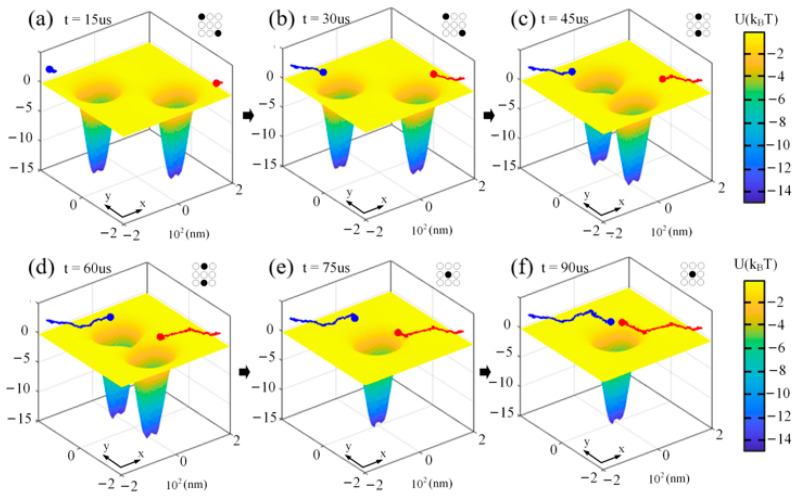
Trajectories of two nanoparticles (blue and red) in a 3 × 3 graphene disk array, and a color map of the trapping potential. The blue line and the red line are the real-time movement tracks. The circular array corresponds to the switching state of the 3 × 3 graphene disc array, the solid circle corresponds to the ON state, and the hollow circle corresponds to the OFF state. (**a**–**f**) are trajectories in different switch configurations and at different times, 15, 30, 45, 60, 75, and 90 us, respectively.

The optical force on the z-axis will make the nanoparticles cling to the partition interface of water and insulator and move in the x-y plane. So we only consider the motion of the nanoparticle in the x-y plane. We choose the electric field intensity at 50 nm above the *z*-axis for the calculation of the force of the optical gradient. We propose here a 3 × 3 array of graphene discs. As mentioned before, the optical forces on the nanoparticles can be switched quickly by controlling the switching states of the adjacent trapping positions. Figure 6 shows the configuration of the graphene disk array at different moments and the corresponding resulting trapping potential, as well as the nanoparticles trajectories. Here the trapping potential is calculated by a simplified method [65]. The trajectory routes of the central positions of the two nanoparticles are marked with two colors (green, red), respectively. The corresponding graphene switching state (Fermi energy) configuration is shown in the top right corner of each figure. Δt = 1 ns, Equation (6) is executed 10^5^ times. The nanoparticles are released at moment 0, and the initial positions are set at (−180 nm, 180 nm), (−180 nm, −180 nm) in the x-y plane (z = 50 nm plane), respectively. As is shown in Figure 6a,b, at the moment of 15 us, the two nanoparticles move to the trapping positions driven by optical forces. The motion of nanoparticles tends to steady-state at t = 30 us. The nanoparticles are stably trapped in two trapping potentials. As is indicated in Figure 6c,d, by controlling the switching state of the graphene disk (Fermi energy), the optical field and the trapping position of the system are readjusted. The nanoparticle moves toward the new trapping position (t = 45 us) and achieves a steady-state at t = 60 us. As is illustrated in Figure 6e,f, the motion of the nanoparticles quickly responded to the change of the electric field, and the blue and red nanoparticles move to the same trapping region at t = 90 us and converged to the steady-state. The trap, transport, and fusion of the nanoparticles are achieved. A larger array of graphene discs can be designed to manipulate the motion of more than two nanoparticles independently.

## 4. Conclusions

In summary, integrated multifunctional graphene discs 2D plasmonic optical tweezers for manipulating nanoparticles is shown to be achievable. We demonstrate that, when the Fermi energy of the graphene disk is 0.6 eV and the incident light wavelength is 8.26 µm, LSPR with compact electric field enhancement is formed. We theoretically calculate the optical forces on the nanoparticles and the trapping potential in the near-field enhancement. The trapping potential generated by the graphene discs in the ON state can cover the adjacent graphene discs. We demonstrate that nanoparticles can be manipulated independently in the x-y plane by modulating the position of the LSPR. Our study shows that the optical force on the nanoparticle is positively correlated with the radius and the relative refractive index of nanoparticles. We investigate the photothermal and thermographic fluidization of our proposed systems, and estimate the forces on the nanoparticles other than the optical force. We consider that the other forces can be neglected under incident intensity I_0_ = 1 mW/µm^2^. Finally, we demonstrate its ability to trap, transport, and fuse nanoparticles with a 3 × 3 array of graphene discs by means of the Langevin equation. We anticipate that it will become a new tool for nanoparticle manipulation and open new directions for its wide range of potential applications in lab-on-chip, nano-assemble, enhanced Raman sensing, etc.

## Figures and Tables

**Table 1:** The components of the force on the nanoparticle.

Force	Method	Maximum (pN)
Optical force	MST	3.25
Brown motion force	$F_{B}=\sqrt{2k_{B}T\gamma }$	2.6126 × 10^3^
Drag force	$F_{D}=\gamma \upsilon$	8.378 × 10^3^
Thermophoretic force	$F_{T}=\gamma D_{T}\nabla T$	7.221 × 10^2^
Gravity force	$F_{g}=\frac{4}{3}\pi r^{3}\rho$	4.974 × 10^3^

## Data Availability

The data that support the findings of this study are available from the corresponding author upon reasonable request.
